# Extensive thoracic spontaneous epidural hematoma presenting with position-induced transient paraplegia: successful conservative management in the resource limited settings

**DOI:** 10.1186/s12245-026-01151-y

**Published:** 2026-02-24

**Authors:** Kotaro Murakami, Noriaki Yamada, Nobuhiro Kodani, Yoshiaki Iwashita

**Affiliations:** 1 Ohda City Hospital, 1428-3 Yoshinaga, Ohda, Shimane 694-0063 Japan; 2 Matsunami General Hospital, 185-1 Dendai, Kasamatsu, Hashima, Gifu, 501-6062 Japan; 3 Yasugi Daiichi Hospital, 899-1 Yasugi, Yasugi, Shimane 692-0011 Japan; 4 Suzuki Internal Medicine and Ophthalmology Clinic, 532-12 Waki, Gotsu, Shimane 695-0017 Japan; 5 Shimane University Hospital, 89-1 Enya-Cho, Izumo, Shimane 693-8501 Japan; 6 Department of Emergency and Critical Care Medicine, Shimane University, Ennyacho 89-1, Izumo, Shimane 693-8501 Japan

**Keywords:** Conservative management, Spontaneous spinal epidural hematoma, Lower extremity paralysis, Posture-dependent neurological symptoms, Thoracolumbar junction

## Abstract

**Background:**

Spontaneous spinal epidural hematoma (SSEH) is a rare condition with an estimated incidence of 0.1 per 100,000 individuals. It is usually characterized by sudden spinal pain followed by rapidly progressive neurological deficits. Surgical treatment is generally indicated if a patient presents with neurological deficits. We report a case of SSEH presenting with neurological deficits relieved after position change, recovering without surgery.

**Case presentation:**

The patient was a 73-year-old woman developed sudden, severe pain in the neck, back, lumbar region, and abdomen while sitting after meals. Upon arrival at the emergency department, her vital signs were stable except for hypertension. The patient was placed supine on a stretcher and became agitated because of back pain, but no motor or sensory symptoms were noted in the extremities. Pain initially improved with acetaminophen but recurred, requiring diclofenac. When attempting to sit again, complete motor and sensory deficits were noted in both lower limbs extending from the femur to the soles of the feet (the trunk was not assessed). Lower limbs muscle strength was 1/1 by Manual Muscle Test. Perineal sensory deficits and incontinence were present, leading to a diagnosis of bladder and bowel dysfunction (ASIA Grade A). Imaging revealed an extensive posterior epidural hematoma from C6 to Th12 with maximal cord compression at Th10–12. No coagulopathy or vascular malformations were observed. The hospital was unable to provide emergency surgical decompression because of limited medical resources, including the unavailability of an on-call spine surgeon and refusal of the transfer request. The patient was managed conservatively with analgesia, blood pressure control, and hemostatic agents. Motor function recovered fully, and she was discharged independently on day 13 with only mild residual sensory impairment.

**Discussion:**

This case demonstrates a unique position-induced course; symptoms improved when supine and worsened while sitting. Treatment of SSEH is generally initiated once neurological symptoms appear. A case report described lumbar SSEH with position-induced symptoms; however, it was the opposite of the present case. This discrepancy reflects differences in position related spinal alignment. Lumbar lordosis decreases while sitting, expanding the canal, whereas thoracic kyphosis flattens when supine and increases while seated, narrowing the canal. In this patient, maximal compression at Th10–12 was relieved when supine but exacerbated when sitting. In this case, conservative treatment was selected due to resource limitations, including the lack of an on-call spine surgeon and refusal of the transfer request. This approach ultimately resulted in the most favorable therapeutic outcome. When rapid improvement in position-induced symptoms is observed, conservative treatment may be a better option. These findings emphasize that SSEH presentation depends not only on hematoma size but also on lesion location and posture-related mechanics.

## Background

Spontaneous spinal epidural hematoma (SSEH) is a rare clinical entity with an estimated incidence of approximately 0.1 per 100,000 individuals [1]. It is typically characterized by a sudden onset of severe spinal pain, frequently followed by rapidly progressive neurological deficits such as paraplegia [2]. Patients with neurological findings, such as paralysis or bladder and bowel dysfunction, are candidates for surgery [3].

We report a case of a 73-year-old woman diagnosed with SSEH. The patient presented with bilateral paralysis during the Emergency Room course, but regained motor function shortly after a posture change.

## Case presentation

The patient was a 73-year-old woman with hypertension who had undergone artificial vascular graft replacement of a left common iliac artery aneurysm 6 months ago. She experienced sudden, severe pain in her neck, back, lumbar region, and abdomen while sitting after a meal at home. She was transported to the emergency department with a blood pressure of 157/94 mmHg, a pulse rate of 70/min, SpO_2_ at 95%, respiratory rate at 24/min, body temperature at 35.5 °C, and Glasgow Coma Scale E3V5M6. The patient was agitated because of back pain while in the supine position on a stretcher, but no motor or sensory symptoms were noted in the extremities.

**Fig. 1:**
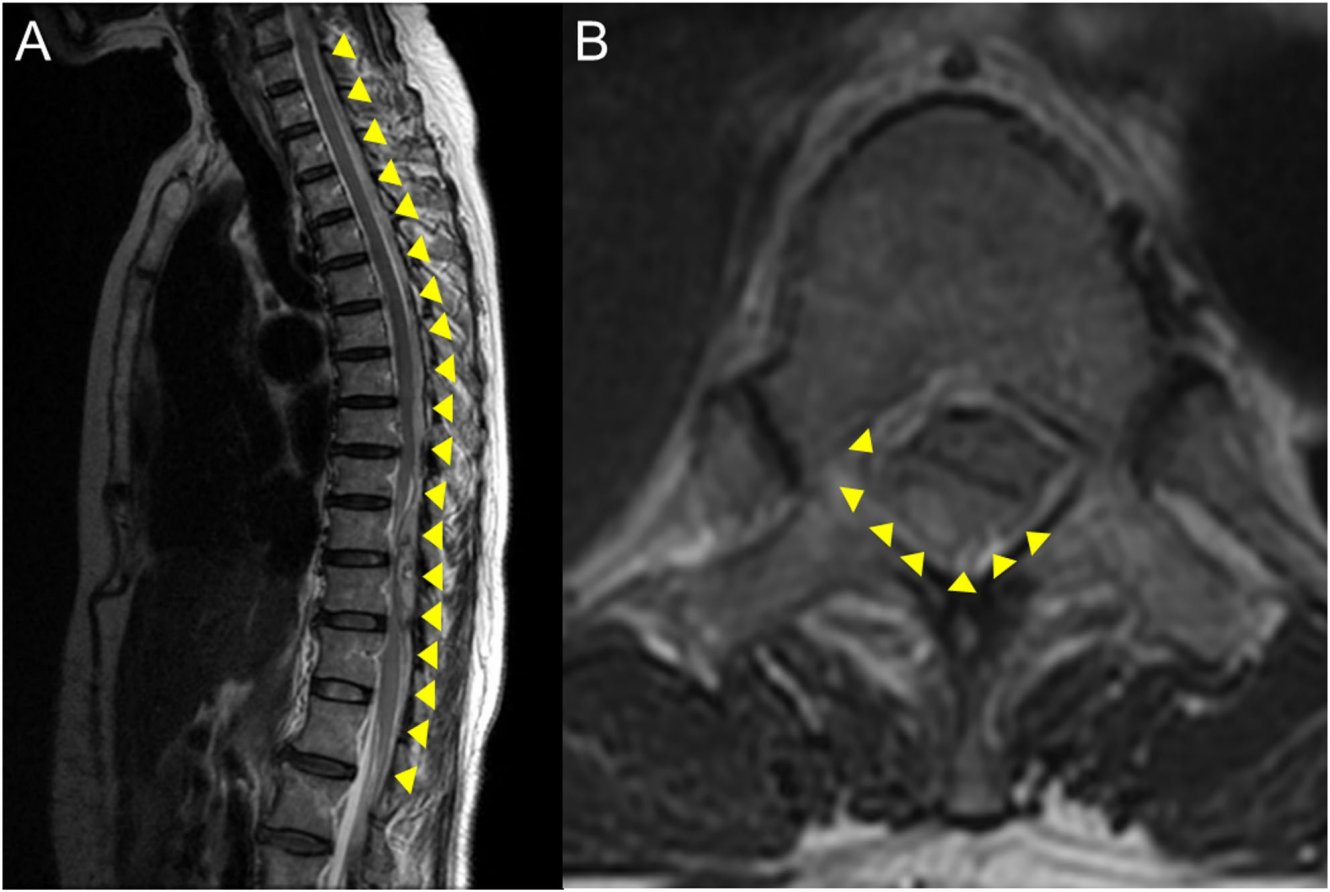
**A**) T2-weighted sagittal magnetic resonance imaging demonstrating a posterior epidural hematoma extending from C6 to Th12. Spinal cord compression is most evident between Th6 and Th12, with maximal compression at Th10–Th12. **B**) T2-weighted axial magnetic resonance imaging at Th11 shows that the hematoma occupied approximately 40% of the spinal canal. The hematoma compressed the spinal cord from the right posterior aspect, resulting in slight flattening of the spinal cord. No abnormal signal intensity was detected within the spinal cord

Acetaminophen 1000 mg was administered intravenously soon after ER arrival, and the pain gradually subsided during contrast computed tomography (CT) imaging approximately 1 h later. After imaging, the pain improved when she remained supine on the stretcher, but recurred upon assuming a sitting position, making the symptoms difficult to control. Upon returning to the supine position, a 50 mg diclofenac suppository was administered, which reduced the lower limbs pain. When attempting to sit again, complete motor and sensory deficits were noted in both lower limbs extending from the femur to the soles of the feet (the trunk was not assessed). There was no pain, and pseudoparalysis was ruled out. Lower limbs muscle strength was 0/0 by Manual Muscle Test. Perineal sensory deficits and incontinence were present, leading to a diagnosis of bladder and bowel dysfunction (ASIA Grade A). Pathological reflexes and tendon reflexes were not assessed. The radiologist diagnosed a spontaneous spinal epidural hematoma (SSEH) extending from the cervical to lumbar spine. During transfer for MRI, her lower limb motor deficit improved when she was placed supine on the stretcher, enabling her to lift her hips. After MRI, the patient’s lower extremity movement recovered to an Manual Muscle Test of 4/4. Mild sensory loss persisted in the lower extremities in the L5 region; however, no motor or bladder or bowel dysfunction were observed. (ASIA Grade B). MRI indicated a posterior hematoma from C6 to Th12, causing spinal cord compression, particularly at Th6–12, with maximum at Th10–12 (Fig. 1). The hematoma measured 9.0 × 6.0 mm in diameter and approximately 300 mm longitudinally. The hematoma occupied approximately 40% of the spinal canal, causing slight spinal cord compression, though no T2-weighted hyperintensity was observed within the cord.

Blood tests showed no coagulopathy, and platelet counts were within normal limits (Table 1).

**Table 1:** Laboratory data

Category	Parameter (Formal Name)	Result	Reference Range
Complete blood count	White blood cell count (WBC, ×10^3^/μL)	11.17	3.3–8.6
	Red blood cell count (RBC, ×10^6^/μL)	4.66	3.86–4.92
	Hemoglobin (Hb, g/dL)	14.6	11.6–14.8
	Hematocrit (Ht, %)	43.6	345.1–44.4
	Mean corpuscular volume (MCV, fL)	93.6	83.6–98.2
	Mean corpuscular hemoglobin concentration (MCHC, g/dL)	33.5	31.7–35.3
	Platelet count (Plt, ×10^4^/μL)	33.1	15.8–34.8
Coagulation	Prothrombin time–international normalized ratio (PT-INR)	0.86	0.9–1.10
	Activated partial thromboplastin time (APTT, sec)	25.6	25.0–39.0
	D-dimer (μg/mL)	1.4	−1
Biochemistry	Total protein (TP, g/dL)	7.4	6.5–8.1
	Albumin (Alb, g/dL)	4.4	4.1–5.1
	Total bilirubin (T-Bil, mg/dL)	0.5	0.4–1.5
	Aspartate aminotransferase (AST, U/L)	24	13–30
	Alanine aminotransferase (ALT, U/L)	17	7–23
	Lactate dehydrogenase (LDH, U/L)	264	124–222
	Creatine kinase (CK, U/L)	92	41–153
	Blood urea nitrogen (BUN, mg/dL)	13.2	8–20
	Creatinine (Cre, mg/dL)	0.55	0.46–0.79
	Sodium (Na, mEq/L)	142	138–145
	Potassium (K, mEq/L)	3.2	3.6–4.8
	Chloride (Cl, mEq/L)	107	101–108
	C-reactive protein (CRP, mg/dL)	0.02	0.00–0.14
	Blood Sugar (BS, mg/dL)	197	73–109

Contrast-enhanced CT and MRI showed no findings suggestive of vascular occlusion or spinal cord infarction, and the mildly elevated D-dimer level was considered a non-specific finding

**Fig. 2:**
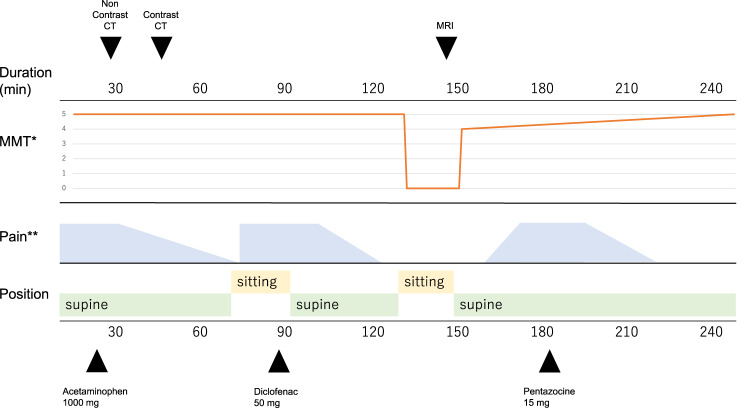
Courses of events after Hospital admission pain and paralysis appeared when sitting and improved when lying down. Occasionally, pain occurred regardless of position. MMT*; Manual muscle testing assessed only hip flexion and extension, and knee flexion and extension. Pain**; Pain was not adequately quantified, and the observer’s subjective assessment was illustrated

Medications included azelnidipine (8 mg), azilsartan (20 mg), and flecainide (50 mg), with no antithrombotic medication. Contrast CT revealed no apparent vascular malformations other than the known aneurysm. The patient was diagnosed with a spontaneous spinal epidural hematoma and was indicated for surgical decompression according to clinical guidelines. The hospital was unable to provide emergency surgical decompression due to lack of an on-call spine surgeon. A transfer request was submitted to a nearby hospital capable of surgical treatment; however, the request was not accepted because of lack of operating room availability. Symptoms were stable and improving in the supine position, and there was no muscle weakness. Imaging studies demonstrated spinal cord compression, but no abnormal intramedullary signal changes were observed. Conservative management with bed rest and blood pressure control was initiated, with plans to reconsider surgical intervention if the symptoms worsened (Figure 2).

Fentanyl and nicardipine were administered for analgesia and blood pressure control, respectively. Prior to symptom onset, systolic blood pressure was elevated at 160–180 mmHg. Management targeted a systolic blood pressure of 140 mmHg. Carbazochrome sodium sulfonate hydrate and tranexamic acid were administered, and the patient remained on bed rest. Although these agents were administered to prevent hematoma enlargement, there is no evidence that carbazochrome sodium sulfonate hydrate or tranexamic acid is effective for SSEH.

The following day, a formal neurological examination was conducted by a spine surgeon. The iliopsoas, quadriceps femoris, hamstrings, tibialis anterior, and gastrocnemius muscles all registered an Manual Muscle Test of 5/5. No neurological deficits were observed in the trunk, and no significant decrease in tactile sensation of the lower limbs was noted. Mild temperature sensation loss was noted in the right lower leg, along with decreased vibration sensation at both patellae and the ankle joints. No decrease or increase in patellar tendon reflexes or Achilles tendon reflexes was observed. No pathological reflexes or decreased perianal sensation were noted (AISA Grade E). Neurological evaluation of the trunk was not adequately performed throughout the course of the case.

**Fig. 3:**
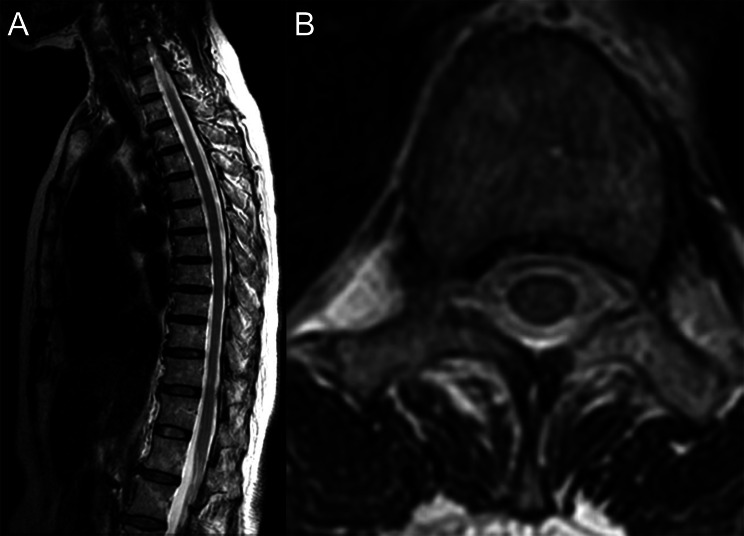
T2-weighted magnetic resonance imaging obtained 7 months after onset shows resolution of the hematoma. **A**) Sagittal section. **B**) Axial section at Th11

On the third day of illness, the patient attempted to sit but experienced pain, so bed rest was continued. No motor or sensory deficits were observed in the supine position, and there was no recurrence of bladder or bowel dysfunction although a formal neuro exam was not performed in the sitting position. On the fifth day of illness, the patient had no pain while receiving fentanyl, and remained pain-free after its discontinuation. MRI also confirmed a reduction in the hematoma (8.1 × 3.9 × 139 mm). Bed rest was discontinued that same day, and the patient was able to stand. On the 13th day of illness, the patient regained independent ambulation and was discharged. At discharge, lower limb muscle strength was 5/5. Sensory deficits in the femur had resolved, with residual deficits confined to the lower legs and feet in the L5 region. Follow-up continued for 7 months after discharge. Sensory deficits remained unchanged, but no paralysis was noted. MRI showed the hematoma had resolved, and follow-up was concluded (Figure 3).

## Discussion and conclusions

We report a rare case of an extensive spinal epidural hematoma extending from the thoracic spine to the thoracolumbar junction. Previous reports have identified antithrombotic therapy and hemodialysis as risk factors for SSEH [4]. The patient had neither undergone hemodialysis nor received antithrombotic drugs. Blood pressure was controlled with antihypertensive medication. Although catheter angiography was not performed, contrast-enhanced CT and MRI showed no vascular structural abnormalities, supporting a diagnosis of spontaneous spinal epidural hematoma. Although external factors such as acupuncture or weight-bearing activities have been reported as triggers [5, 6], the patient had no such history. Our patient demonstrated alleviation of symptoms while supine, and neurological deterioration upon assuming a seated posture. Conversely, previous reports described lumbar spinal epidural hematomas with opposite clinical courses, characterized by symptom exacerbation in the supine position and improvement while sitting [7].

Only one previous report described position-induced symptom variation. The reverse clinical course—characterized by symptom worsening in the supine position and improvement in the sitting position—has been documented in lumbar spinal epidural hematoma [7]. Symptom relief in the sitting position was attributed to the gravitational redistribution of blood. Multiple cases have described transient symptom exacerbation unrelated to position [2, 8–11], with a similar hypothesis attributing this to blood redistribution. However, in this case, symptoms improved in the sitting position, suggesting that additional factors should be considered. Changes in position may alter epidural venous pressure, potentially causing symptom fluctuations. The epidural venous plexus lacks valves [12], and thoracic epidural venous pressure is likely to increase while lumbar epidural venous pressure decreases compared with the supine position. Therefore, if this hypothesis is correct, neurological symptoms may worsen in the sitting position for lumbar lesions and in the supine position for thoracic lesions. However, in practice, symptoms worsen in the supine position for lumbar lesions and in the sitting position for thoracic lesions. Thus, changes in epidural venous pressure cannot explain this pattern. Other factors include the relatively poor blood flow to the middle thoracic spinal cord. Transient blood pressure drops caused by sitting may also exacerbate neurological symptoms. However, in this case, pain sometimes occurs during sitting, suggesting the effect is more likely due to compression than blood flow. This divergence likely reflects the distinct biomechanical effects of posture on spinal alignment in the thoracic and lumbar regions. Previous studies have established that lumbar lordosis remains nearly unchanged between standing and supine postures but decreases markedly while sitting [13]. It is also known that the anteroposterior diameter of the dural sac shortens in the supine position and lengthens in the sitting position [14]. These factors may reduce compression on the lumbar spine when sitting. Conversely, thoracic kyphosis decreases (flattens) in the supine position and increases during relaxed sitting compared with erect sitting [15, 16]. In the present case, maximal compression occurred at Th10–12 in the lower thoracic spine. We postulate that, in the supine position, reduced thoracic kyphosis transiently increases the anteroposterior diameter of the spinal canal, mitigating cord compression. Alternatively, in the sitting position, the augmentation of thoracic kyphosis likely resulted in the narrowing of the spinal canal at the affected levels, resulting in abrupt bilateral lower-extremity paralysis.

These observations underscore that the clinical manifestations of spinal epidural hematoma are determined not only by hematoma size but also by the interaction between lesion location and position related spinal alignment. As posture-related variations in the spinal canal diameter differ between the thoracic and lumbar segments, the pattern of symptom onset and fluctuation may vary accordingly.

The limitation of this case is that paralysis was observed only once. After complete motor and sensory loss of the lower limbs appeared in the sitting position, the patient was kept in a supine position to avoid further neurological damage. On the third day of illness, pain was reproduced in the sitting position, but paralysis was not. Therefore, it cannot be concluded that complete motor and sensory loss of the lower limbs resulted solely from changes in position. Multiple factors are thought to be involved.

Surgical intervention for SEH is widely recognized as the standard approach. Symptoms persisting for more than 24 hours are considered to indicate a poor prognosis, and those lasting more than 24 hours require active surgical treatment [17, 18]. Surgery may not be necessary if neurological symptoms improve rapidly [3, 8, 19]. This case presented with complete motor and sensory loss while sitting, which generally indicates a need for surgical treatment. Due to the symptom course, environmental factors, and the surgical risks associated with advanced age, conservative treatment was selected, and this approach ultimately resulted in the most favorable therapeutic outcome. When rapid improvement in position-indeced symptoms is observed, conservative treatment may be a better option. Our case emphasizes the need to consider the anatomical level of the hematoma and the mechanical consequences of position when evaluating and managing patients with spinal epidural hematomas.

## Data Availability

Not applicable.

## Abbreviations

SSEH: Spontaneous spinal epidural hematoma
ED: Emergency department
CT: Computed tomography
MRI: Magnetic resonance imaging
SEH: Spinal epidural hematoma
